# 0728. Removal of natural anti-galactose α1,3 galactose antibodies with GAS914 enhances humoral immunity and prevents sepsis mortality in mice

**DOI:** 10.1186/2197-425X-2-S1-P50

**Published:** 2014-09-26

**Authors:** R Manez, M Perez-Cruz, D Bello, MA Dominguez, C Costa

**Affiliations:** Intensive Care Medicine, Hospitalet de Llobregat, Hospital Universitari de Bellvitge, Spain; Hospitalet de Llobregat, Idibell, Hospital Universitari de Bellvitge, Spain; Hospitalet de Llobregat, Hospital Universitari de Bellvitge, Spain

## Introduction

Increasing numbers of patients with immunodeficiencies, malignant diseases, advanced surgeries and prolonged stays in intensive care units are at risk for developing infections by enteric bacteria, which often are systemic (sepsis) and caused by multidrug resistance isolates. Intestinal bacteria provide also a constant antigenic stimulation for the synthesis of natural anti-carbohydrate antibodies such as those targeting blood group-associated antigens or galactose α1,3 galactose (Gal). Enteric bacteria that cause sepsis are more likely to bind anti-Gal antibodies than those isolated from stools, which appears related to the higher resistance to serum killing evidenced by the former microorganisms.

## Objective

Investigate the impact of removing anti-Gal antibodies in the sepsis after cecal ligation and puncture (CLP) in α1,3 galactosyltransferase knockout (Gal-KO) mice. These mice produce from four months of age natural anti-Gal antibodies as humans without the need of any additional Gal antigen exposure.

## Methods

Anti-Gal antibodies were removed *in vivo* using a soluble Gal trisaccharide-polylysine conjugate (GAS914) and determined by ELISA. Binding of serum antibodies to *Escherichia Coli* isolated from Gal-KO mice blood was performed by FACS and serum bactericidal activity was determined by a complement-mediated assay. The pattern of 40 cytokines and antibodies against 577 carbohydrate and bacterial antigens before and after GAS914 was evaluated by two different arrays.

## Results

GAS914 at 10 mg/kg subcutaneously every other day for two weeks led to the removal of anti-Gal antibodies in Gal-KO mice from day one. Removal of anti-Gal antibodies with GAS914 significantly improved survival in Gal-KO mice subsequently subjected to CLP on day 3 (83% versus 35% in controls; p< 0.01). On day one after CLP there was a reduction of 50% in the level of anti-Gal antibodies in untreated Gal-KO mice. Blood cultures obtained 24 h after CLP were positive for *Escherichia Coli* in 83% of mice carrying anti-Gal antibodies and 87.5% of the animals lacking them. Pulse field electrophoresis did not show differences between *E. Coli* blood isolates from GAS914-treated and untreated mice. Removal of anti-Gal antibodies was associated with a significant increase in serum IgG binding and bactericidal activity to *E. Coli*. GAS914 administration was associated in the glycan array with the removal of anti-Gal antibodies and the significant augment of binding to several bacterial antigens. Finally, GAS914 also significantly reduced the level of several proinflammatory and anti-inflammatory cytokines prior to CLP in Gal-KO mice.

## Conclusion

Removal of natural anti-Gal antibodies with GAS914 enhances humoral immunity against enteric bacteria expressing Gal-antigen isolated from the blood preventing sepsis mortality after CLP in mice. Depletion of existing antibodies may be a new approach to boost immunity and prevent or treat infection diseases and other disorders.

